# JFK Is a Hypoxia-Inducible Gene That Functions to Promote Breast Carcinogenesis

**DOI:** 10.3389/fcell.2021.686737

**Published:** 2021-07-15

**Authors:** Ziran Yang, Xuehong Zhou, Enrun Zheng, Yizhou Wang, Xinhua Liu, Yue Wang, Yanpu Wang, Zhaofei Liu, Fei Pei, Yue Zhang, Jie Ren, Yunchao Huang, Lu Xia, Sudun Guan, Sen Qin, Feiya Suo, Jie Shi, Lijing Wang, Lin He, Luyang Sun

**Affiliations:** ^1^Department of Biochemistry and Molecular Biology, School of Basic Medical Sciences, Key Laboratory of Carcinogenesis and Translational Research (Ministry of Education), Peking University Health Science Center, Beijing, China; ^2^Department of Biochemistry and Molecular Biology, School of Basic Medical Sciences, Hangzhou Normal University, Hangzhou, China; ^3^Medical Isotopes Research Center and Department of Radiation Medicine, School of Basic Medical Sciences, Peking University Health Science Center, Beijing, China; ^4^Department of Pathology, Peking University Third Hospital, School of Basic Medical Sciences, Peking University Health Science Center, Beijing, China; ^5^National Institute on Drug Dependence, Peking University, Beijing, China; ^6^Vascular Biology Research Institute, Guangdong Pharmaceutical University, Guangzhou, China; ^7^Department of Integration of Chinese and Western Medicine, School of Basic Medical Sciences, Peking University Health Science Center, Beijing, China

**Keywords:** breast carcinogenesis, transcriptional regulation, cancer metabolism, hypoxia, JFK

## Abstract

Many carcinomas feature hypoxia, a condition has long been associated with tumor progression and poor prognosis, as well as resistance to chemoradiotherapy. Here, we report that the F-box protein JFK promotes mammary tumor initiation and progression in MMTV-PyMT murine model of spontaneous breast cancer. We find that *JFK* is inducible under hypoxic conditions, in which hypoxia-inducible factor HIF-1α binds to and transcriptionally activates *JFK* in breast cancer cells. Consistently, analysis of public clinical datasets reveals that the mRNA level of JFK is positively correlated with that of HIF-1α in breast cancer. We show that JFK deficiency leads to a decrease in HIF-1α-induced glycolysis in breast cancer and sensitizes hypoxic breast cancer cells to ionizing radiation and chemotherapeutic treatment. These results indicate that JFK is an important player in hypoxic response, supporting the pursuit of JFK as a potential therapeutic target for breast cancer intervention.

## Introduction

Among females, breast cancer is the most commonly diagnosed cancer and the leading cause of cancer death (Bray et al., 2018). Although the development of comprehensive breast cancer treatment strategies has supported a downward trend in breast cancer mortality, there are still no means of definitively identifying patients who will be resistance to radiotherapy and chemotherapy and relapse (Akram et al., 2017). Evidence is mounting that the oxygen (O_2_) content of tumor tissue is an important determinant of metastasis, and tumor hypoxia has been associated with increased malignancy, poor prognosis, and resistance to both radiotherapy and chemotherapy (Keith et al., 2011). Thus, particular interest has been focused on the mechanisms by which hypoxic tumor cells alter their transcriptional profiles to modulate processes including initiation, progression, and metastasis to persist under conditions of hypoxic stress.

Aerobic glycolysis, also termed the Warburg effect, is a general feature of glucose metabolism in tumor cells (Hanahan and Weinberg, 2011; Chen et al., 2019). Tumor cells predominantly utilize glycolysis for energy production mechanisms and have higher rates of glycolysis, which increases biosynthesis, inhibits apoptosis, and generates signaling metabolites to enhance tumor cell survival under difficult conditions including hypoxia (Pavlova and Thompson, 2016; Wu et al., 2020). It is well established that hypoxia can lead to high rates of glycolysis through stabilization of hypoxia-inducible factors (HIFs) (Wu et al., 2020). HIFs are heterodimeric complexes composed of basic helix-loop-helix/PAS (bHLH-PAS) proteins including an O_2_-labile alpha subunit (HIF-1α, HIF-2α, or HIF-3α) and a stable beta subunit (HIF-1β) (Iommarini et al., 2017). HIF transcription factors are master regulators of the cellular response to hypoxia and coordinate a transcriptional program that ensures optimal functional, metabolic, and vascular adaptation to oxygen shortages (Jurikova et al., 2016). In breast cancer, HIF-1α transcriptionally activates the expression of genes with known functions in cell survival, angiogenesis, metabolic reprogramming, invasion, and metastasis, through binding to hypoxia response elements (HREs) in the promoters of its target genes (Masoud and Li, 2015). However, it remains to be investigated the mechanism(s) underlying how cancer cells response to hypoxic conditions through HIF-1α-mediated transcriptional regulation.

To date, the best-characterized mammalian multi-subunit RING-finger type of E3 ligases is the SCF (SKP1-CUL1-F-box) complex, comprising SKP1, RBX1, CUL1, and a flexible F-box protein (FBP) that acts as a receptor for substrate recognition (Jia and Sun, 2011). In previous studies, we reported that JFK is the only Kelch domain-containing F-box protein in humans, and targets p53 and ING4 for degradation through the assembly of a SCF complex (Sun et al., 2009, 2011; Yan et al., 2015). We showed that JFK destabilizes p53, contributing to reduced cell apoptosis and sensitizing cells to ionizing radiation-induced cell death. Additionally, JFK-mediated ING4 proteasomal degradation promotes angiogenesis and metastasis of breast cancer. JFK is highly expressed in breast cancer, supporting that it could be used as a target molecule for the design of new breast cancer therapies. However, the upstream activators (and cellular contexts) which drive elevated JFK levels in breast cancers are currently unknown.

In the present study, using MMTV-PyMT model of spontaneous breast cancer, we report that JFK promotes mammary tumorigenesis. HIF-1α binds to and transcriptionally activates *JFK* in hypoxic breast cancer cells, and analyses of public clinical datasets support the positive correlation between mRNA expression of JFK and HIF-1α in breast cancer. We showed that JFK knockdown leads to a decrease in hypoxia-enhanced glycolysis and overcomes chemo-radiotherapeutic resistance in hypoxic breast cancer cells. We demonstrated that *JFK* is a hypoxia-induced oncogene, and explored the clinicopathological significance of the HIF-1α-JFK axis in breast cancer progression and intervention.

## Materials and Methods

### Antibodies

The antibodies used were αTubulin from Sigma; αHIF-1α from Abcam; αp53, αING4, αKi-67, αPCNA, αE2F1, αFOXP3, and αMYC from Santa Cruz. Polyclonal antibodies against JFK were raised against the C-terminal epitope of the JFK protein (CYPKTNALYFVRAKR) in rabbits.

### *JFK* Transgenic Mice

*JFK* transgenic mice were generated by Cyagen Biosciences on the C57BL/6 background. ORF of human *JFK* with a FLAG tag was cloned into the mammalian expression vector pRP (Exp)-EF1A. After digestion, linearized DNA was used for microinjection into the pro-nuclei of fertilized oocytes from hormonally superovulated C57BL/6 female mice under a microscope. The injected fertilized eggs were transplanted into the oviducts of pseudo-pregnant mice. The genomic DNA were extracted from mouse tail tips for molecular genotyping. The primers specific for the transgene *JFK* (forward: 5′-GCTTTTGGAGTACGTCGTCT-3′; reverse: 5′-GGCTCCTCATCTTGATCCAT-3′) were used to amplify a 334 bp fragment, and the primers for internal control (forward: 5′-CAACCACTTACAAGAGACCCGTA-3′; reverse: 5′-GAGCCCTTAGAAATAACGTTCACC-3′) were used to amplify a 632 bp fragment.

### MMTV-PyMT Mouse Model

FVB/N-Tg (MMTV-PyMT) mice were crossed with wild-type mice (C57BL/6 strain) for six generations to backcross into the C57BL/6 strain. Genotyping analyses were performed by PCR with genomic DNA extracted from tail tips. The primers specific for the transgene *PyMT* (forward: 5′-GGAAGCAAGTACTTCACAAGGG-3′; reverse: 5′-GGAAAGTCACTAGGAGCAGGG-3′) were used to amplify a 556 bp fragment. Animal handling and procedures were approved by the Institutional Animal Care of Peking University Health Center.

### Small-Animal SPECT/CT Imaging of ^99m^Tc-3PRGD2

The integrin αvβ3-targeting radiotracer ^99m^Tc-3PRGD2 was synthesized by labeling the precursor HYNIC-3PRGD2 with Na^99m^TcO_4_ as previously described (Jia et al., 2011). ^99m^Tc-3PRGD2 was prepared with the radiochemical purity of >98% and was then used for *in vivo* imaging studies. For small-animal SPECT/CT, MMTV-PyMT mice were intravenously injected with 37 MBq of ^99m^Tc-3PRGD2. At 30 min postinjection, SPECT/CT scanning was performed using a NanoScan SPECT/CT Imaging System (Mediso, Budapest, Hungary) as previously described (Liu et al., 2012, 2014).

### Cell Culture and Transfection

MCF-7 and T47D cells were maintained according to the ATCC’s recommendation. Transfections of expression plasmids were carried out using polyethyleneimine (PEI, Polysciences), and siRNA oligonucleotides (GeneChem Inc.) were transfected into cells using RNAiMAX (Invitrogen) according to the manufacturer’s instructions. The sequences of siRNAs were: HIF-1α siRNA, 5′-UUCAAGUUGGAAUUGGUAG-3′; JFK siRNA, 5′-GGUGUAGCCCAUCAGUGUU-3′; and control siRNA, 5′-UUCUCCGAACGUGUCACGU-3′.

### Western Blotting

Cellular lysates were prepared by incubating the cells in lysis buffer (50 mM Tris–HCl, pH 7.5, 150 mM NaCl, 0.5% NP-40, and 2 mM EDTA) containing protease inhibitor cocktail and phosphatase inhibitor for 20 min at 4°C, followed by centrifugation at 14,000 *g* for 15 min at 4°C. The protein concentration of the lysates was determined using the BCA protein assay kit (Pierce) according to the manufacturer’s protocol. For western blotting, the samples were resolved on SDS-PAGE gels and transferred onto nitrocellulose membranes. The membranes were incubated with appropriate antibodies overnight at 4°C followed by incubation with a secondary antibody. Immunoreactive bands were visualized using western blotting Luminol reagent (Santa Cruz) according to the manufacturer’s recommendation. The dilutions of primary antibodies were: 1: 1000 with αJFK, αING4, αKi-67, αPCNA, or αHIF-1α; 1: 2000 with αp53 or αTubulin.

### Real-Time Reverse Transcription PCR

Total mRNAs were isolated with Trizol reagents (Invitrogen) for cDNA synthesis. Real-time reverse transcriptase PCR was performed using the ABI PRISM 7500 system (Applied Biosystems) that measures real-time SYBR green fluorescence and then calculated by means of the comparative Ct method (2-ΔΔCt) with the expression of GAPDH as an internal control. The primers used were: *JFK*: 5′-AGCCCCACCCAGTATTGGA-3′ (forward) and 5′-CCTGATACGGTGAGAGAAAGGA-3′ (reverse); *GAPDH:* 5′-GAAGGTGAAGGTCGGAGTC-3′ (forward) and 5′-GAAGATGGTGATGGGATTTC-3′ (reverse).

### ChIP and qChIP

MCF-7 or T47D cells treated with hypoxia (1% O_2_) for 24 h were crosslinked with 1% formaldehyde for 10 min at room temperature and quenched by the addition of glycine to a final concentration of 125 mM for 5 min. The fixed cells were resuspended in lysis buffer (1% SDS, 5 mM EDTA, and 50 mM Tris–HCl, pH 8.1) containing protease inhibitors, then subjected to 30 cycles (30 s on and off) of sonication (Bioruptor, Diagenode) to generate chromatin fragments of ∼300 bp in length. Lysates were diluted in buffer (1% Triton X-100, 2 mM EDTA, 150 mM NaCl, and 20 mM Tris–HCl, pH 8.1) containing protease inhibitors. For immunoprecipitation, the diluted chromatin was incubated with normal IgG (control) or HIF-1α antibodies overnight at 4°C with constant rotation, followed by incubation with 50 μl of 50% (v/v) protein A/G Sepharose beads for an additional 2 h. Beads were successively washed with the following buffers: TSE I (0.1% SDS, 1% Triton X-100, 2 mM EDTA, 150 mM NaCl, and 20 mM Tris–HCl, pH 8.0); TSE II (0.1% SDS, 1% Triton X-100, 2 mM EDTA, 500 mM NaCl, and 20 mM Tris–HCl, pH 8.0); TSE III (0.25 M LiCl, 1% Nonidet P-40, 1% sodium deoxycholate, 1 mM EDTA, and 10 mM Tris–HCl, pH 8.0). The pulled-down chromatin complex eluted by TE (1 mM EDTA and 10 mM Tris–HCl, pH 8.0) and input were de-crosslinked at 55°C for 12 h in elution buffer (1% SDS and 0.1 M NaHCO_3_) (Li et al., 2016). The DNA was purified with the QIAquick PCR Purification Kit (QIAGEN). qChIPs were performed using Power SYBR Green PCR Master Mix and an ABI PRISM 7500 system (Applied Biosystems, Foster City, CA, United States). The primers used were: *JFK*: 5′-TGAGCACATTTGTGTGCCG-3′ (forward) and 5′-TGGACACTACTAGGCGGTCA-3′ (reverse).

### Luciferase Reporter Assay

MCF-7 or T47D cells in 24-well plates were infected with lentiviruses carrying luciferase reporter, renilla, and indicated expression constructs. The amount of DNA in each transfection was kept constant by addition of empty vector. Twenty-four hours after transfection, the firefly and renilla luciferase were assayed according to the manufacturer’s protocol (Promega), and the firefly luciferase activity was normalized to that of renilla luciferase. Each experiment was performed in triplicate and repeated at last three times.

### Measurement of Glucose Uptake and Lactate Production

The extracellular lactate was measured using the cell culture medium with Lactate Colorimetric Assay Kit (BioVision) according to the manufacturer’s instruction. Intracellular glucose was measured using cell lysates with Glucose Colorimetric/Fluorometric Assay Kit (BioVision) according to the manufacturer’s instruction.

### Histologic Analysis

Mammary tumors or livers of female mice were excised, fixed with 10% neutral buffered formalin for 24 h. After paraffin embedding and sectioning (5 μm), tissues were stained with hematoxylin and eosin (H&E). In tissue immunohistochemical staining, the antigen was retrieved by high pressure and incubation in 0.01 M sodium citrate buffer. Then the samples were blocked in 10% normal goat serum in PBS, and incubated with primary antibodies at 4°C overnight in primary antibody solution. After being washed with PBS buffer, the samples were incubated with polymer HRP goat anti-rabbit (DAKO, Agilent) for 30 min at room temperature, developed with DAB (3,3′-diaminobenzidine tetrahydrochloride), and counterstained with hematoxylin (Zhongshan Golden Bridge Biotechnology Company). All specimens were evaluated by two pathologists without knowledge of the experimental group.

### Cell Viability Assay

MCF-7 cells with control or JFK-depleted were cultured in normoxia or hypoxia for 48 h, and were subsequently treated with X-ray irradiation, tamoxifen, or fulvestrant before growing for 5 days. The cell viability rate of MCF-7 cells was evaluated using Cell Counting Kit (CCK, TransGen Biotech) according to the manufacturer’s instruction.

### Statistical Analysis

Results are reported as mean ± SD for triplicate experiments or mean ± SEM unless otherwise noted. SPSS V.19.0 was used for statistical analysis. Comparisons between two groups were performed using two-tailed paired Student’s *t* test. Comparisons among three groups were performed using one-way ANOVA followed by Tukey’s honestly significant difference (HSD) *post hoc* test.

## Results

### JFK Promotes Mammary Tumor Initiation and Metastasis in Mice

It has been reported that JFK is highly expressed in tumor tissues including breast, kidney, and pancreatic cancer, with the highest JFK expression level reported in breast cancer (Yan et al., 2015). To further investigate the potential function of JFK in breast cancer development, we first generated *JFK* transgenic (*JFK*^*TG*^) mice by nuclear transplantation. Briefly, the open reading frame (ORF) of human *JFK* (with a FLAG tag) was cloned into the mammalian expression vector pRP(Exp)-EF1A. After digestion, linearized DNA was micro-injected into the pronuclei of fertilized zygotes derived from C57BL/6 female mice, and the embryos were transplanted onto pseudo-pregnant mice to breed *JFK*^*TG*^ founders.

**FIGURE 1:**
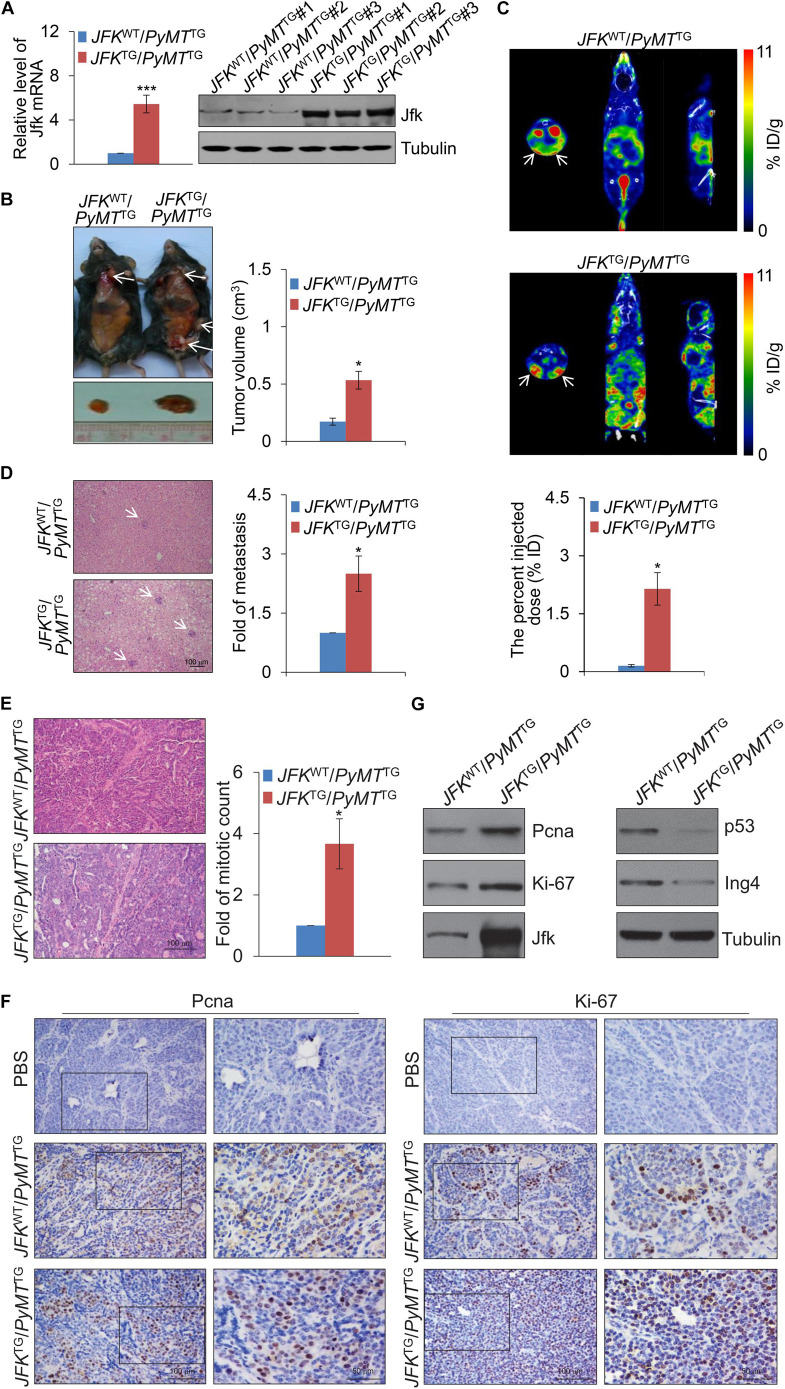
JFK promotes mammary tumor initiation and metastasis in mice. **(A)** Total RNAs or proteins from the tail tip of *JFK*^*WT*^/*PyMT*^*TG*^ or *JFK*^*TG*^/*PyMT*^*TG*^ mice were extracted, and respectively, analyzed for Jfk expression by qPCR or western blotting with the indicated antibodies. Error bars represent the mean ± SD for triplicate experiments (***p < 0.001). **(B)** Representative images of tumors resected from 14-week-old *JFK*^*WT*^/*PyMT*^*TG*^ or *JFK*^*TG*^/*PyMT*^*TG*^ mice on autopsy. Arrows indicate the subcutaneous breast tumors, and tumor size was measured using digital calipers. Error bars represent the mean ± SEM (*n* = 6, *p < 0.05). **(C)** Representative SPECT/CT images of *JFK*^*WT*^/*PyMT*^*TG*^ or *JFK*^*TG*^/*PyMT*^*TG*^ mice at 30 min after injection of ^99^mTc-3PRGD2 (37 MBq) via the tail vein. Error bars represent the mean ± SEM (*n* = 6, *p < 0.05). **(D)** Representative images of liver sections from *JFK*^*WT*^/*PyMT*^*TG*^ or *JFK*^*TG*^/*PyMT*^*TG*^ mice stained with hematoxylin and eosin (H&E) are shown. Bar, 100 μm. Error bars represent the mean ± SEM (*n* = 6, *p < 0.05). **(E)** Representative images of tumor sections from 14-week-old *JFK*^*WT*^/*PyMT*^*TG*^ and *JFK*^*TG*^/*PyMT*^*TG*^ mice stained with H&E. Bar, 100 μm. Error bars represent the mean ± SEM (*n* = 6, *p < 0.05). **(F)** Immunohistochemical staining of mammary tumors with from 14-week-old *JFK*^*WT*^/*PyMT*^*TG*^ and *JFK*^*TG*^/*PyMT*^*TG*^ mice for the expression of Ki-67 and Pcna. **(G)** Total protein extracts were prepared from mammary tumor tissues from 14-week-old *JFK*^*WT*^/*PyMT*^*TG*^ and *JFK*^*TG*^/*PyMT*^*TG*^ mice and analyzed via western blotting with the indicated antibodies.

The expression of mRNA and protein of JFK in *JFK*^*TG*^ mice was verified by real-time reverse transcriptase PCR (qPCR) and western blotting, and these both indicated that JFK transgenic mice were obtained successfully (Figure 1A). We then crossed the *JFK*^*TG*^ mice with mouse mammary tumor virus-polyoma virus middle T-antigen (MMTV-PyMT) transgenic mice (Murai, 2015) – a widely utilized preclinical mouse model that recapitulates human breast cancer progression from early hyperplasia to malignant breast carcinoma – to obtain female *JFK*^*WT*^/*PyMT*^*TG*^ and *JFK*^*TG*^/*PyMT*^*TG*^ mice. We observed that *JFK*^*TG*^/*PyMT*^*TG*^ mice developed palpable tumors at the average age of 12-week-old, 2 weeks earlier than *JFK*^*WT*^/*PyMT*^*TG*^ mice. Moreover, the number of subcutaneous tumors was significantly greater, and the volume of subcutaneous tumors was significantly larger in *JFK*^*TG*^/*PyMT*^*TG*^ mice compared with *JFK*^*WT*^/*PyMT*^*TG*^ mice (Figure 1B).

Previous studies reported that integrin α_*v*_β_3_ is expressed at high levels on the surface of a variety of solid tumor cells and on the tumor vasculature (Liu et al., 2011). To further explore the effect of JFK on breast carcinogenesis, we used an integrin α_*v*_β_3_-targeting radiotracer, ^99m^Tc-3PRGD2 (Zhu et al., 2012), to conduct single photon emission computed tomography (SPECT)/computed tomography (CT) *in vivo* imaging. To this end, ^99m^Tc-3PRGD2 was injected into the 14-week-old *JFK*^*WT*^/*PyMT*^*TG*^ and *JFK*^*TG*^/*PyMT*^*TG*^ mice via the tail vein. SPECT/CT of *JFK*^*WT*^/*PyMT*^*TG*^ and *JFK*^*TG*^/*PyMT*^*TG*^ mice at 30 min after injection showed that there was remarkable accumulation of ^99m^Tc-3PRGD2 in mammary tumors at 30 min post-injection, and *JFK*^*TG*^/*PyMT*^*TG*^ mice developed more multifocal palpable tumors compared with *JFK*^*WT*^/*PyMT*^*TG*^ mice (Figure 1C and Supplementary Movies 1, 2). The quantification analysis of total tumor volumes revealed that *JFK*^*TG*^/*PyMT*^*TG*^ mice exhibited increased tumorigenesis compared with *JFK*^*WT*^/*PyMT*^*TG*^ mice (Figure 1C). Hematoxylin-eosin (H&E) staining analysis of liver tissues also revealed that the *JFK*^*TG*^/*PyMT*^*TG*^ mice had significantly elevated numbers of liver metastases compared to *JFK*^*WT*^/*PyMT*^*TG*^ mice (Figure 1D). Collectively, these *in vivo* results support that JFK overexpression somehow promotes mammary tumor initiation and metastasis.

To further explore the role of JFK in promoting breast cancer, we collected breast tumors from 14-week-old *JFK*^*WT*^/*PyMT*^*TG*^ and *JFK*^*TG*^/*PyMT*^*TG*^ mice and performed histological analysis. H&E staining of tumor sections showed that abnormal mitoses in mammary tumor tissue of *JFK*^*TG*^/*PyMT*^*TG*^ mice are about 3-fold greater in number than in *JFK*^*WT*^/*PyMT*^*TG*^ mice (Figure 1E), clearly suggesting that JFK overexpression somehow promotes cell division of breast cancer cells *in vivo*. Pursuing this, we performed immunohistochemical analysis of breast tumor tissues of *JFK*^*WT*^/*PyMT*^*TG*^ and *JFK*^*TG*^/*PyMT*^*TG*^ mice for expression of Ki-67 and Pcna, two well-documented markers for cellular proliferation (Jurikova et al., 2016). *JFK*^*TG*^/*PyMT*^*TG*^ mice exhibited substantially more Ki-67-positive and Pcna-positive nuclei than *JFK*^*WT*^/*PyMT*^*TG*^ mice (Figure 1F), indicating that JFK promotes cell proliferation *in vivo*. Western blotting analysis showed that overexpression of JFK was accompanied by increased accumulation of the Ki-67 and Pcna proteins but reduced levels of the p53 and Ing4 proteins, findings consistent with previous studies showing that JFK targets p53 and ING4 proteins for ubiquitination and degradation (Sun et al., 2009; Yan et al., 2015; Figure 1G) and supporting the aforementioned hypothesis that JFK impacts tumor progression through its function in SCF^*JFK*^ in destabilizing tumor suppressor genes. Together, these *in vivo* results show that *JFK* functions in mammary tumorigenesis in the MMTV-PyMT mouse model by promoting cell proliferation.

### Genome-Wide Identification of Transcriptional Targets of HIF-1α

**FIGURE 2:**
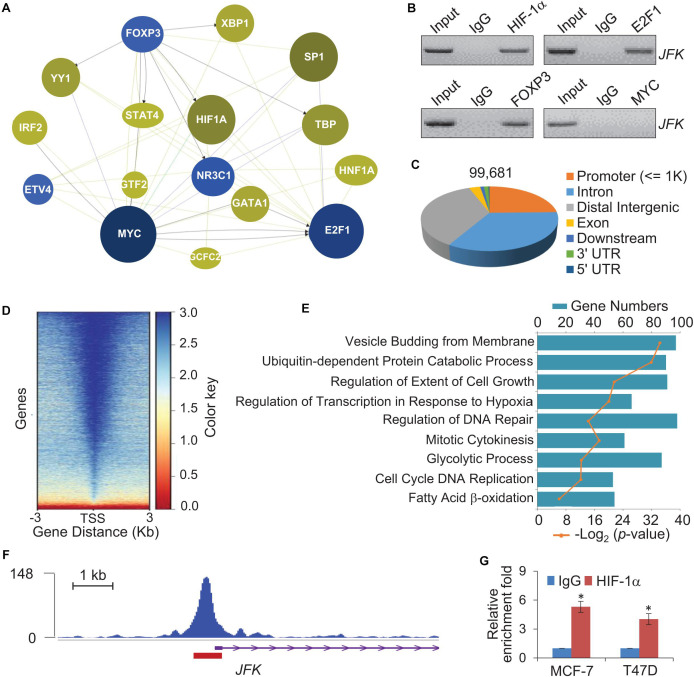
Genome-wide identification of transcriptional targets for HIF-1α. **(A)** Breast cancer associated transcription factors which bind at the promoter of *JFK* were assessed using a Phenolyzer approach. The size and shade of each circle represent the strength of correlation between a given transcription factor that was predicted to bind at the promoter of *JFK* and breast cancer. **(B)** Soluble chromatin from MCF-7 cells was prepared for ChIP assays with antibodies against the indicated proteins. **(C)** Genomic distribution of HIF-1α determined by ChIP-seq analysis. ChIP was performed in hypoxia-treated T47D cells with antibodies against HIF-1α, followed by deep sequencing. The raw data were analyzed by MACS2 with a cutoff *q* value < 1e-2. **(D)** Binding density of HIF-1α across its specific binding sites, visualized by deepTools. The heatmap shows the normalized ChIP-seq tag counts ordered by signal strength. **(E)** GO enrichment (biological processes) analysis of potential target genes. **(F)** Tracks visualizing the ChIP signal for HIF-1α binding at representative locus. The red rectangle indicates the peak region/binding site of HIF-1α at the *JFK* promoter. **(G)** qChIP-based verification of HIF-1α binding at the *JFK* locus under hypoxia. Soluble chromatin from MCF-7 or T47D cells exposed with hypoxia (1% O_2_) for 24 h was prepared, and qChIP analysis was performed for the *JFK* promoter using the antibody against HIF-1α. Error bars represent the mean ± SD for triplicate experiments. Statistical significance of differences is indicated as *p < 0.05.

Given that JFK is expressed at aberrantly high levels in breast cancer at both the mRNA and protein levels, we next explored mechanism(s) underlying JFK dysregulation in diverse samples by adopting a phenolyzer approach (Yang H. et al., 2015). Specifically, to predict any transcription factors likely to bind at the *JFK* promoter in breast cancer, we analyzed data from PROMO^1^ using tools for the phenotype-based gene analyzer^2^. We initially focused on transcription factors with reported links to breast cancer, and found that 16 transcription factors including HIF-1α, MYC, FOXP3, and E2F1 were predicted to target the *JFK* locus (Figure 2A). Soluble chromatin from MCF-7 cells was prepared for ChIP assays, and the results showed that HIF-1α, FOXP3, and E2F1 were enriched at *JFK* promoter (Figure 2B). Briefly, HIF-1α overexpression, but not FOXP3 nor E2F1, has been reported to be closely related to high histological grade, lymph node metastasis, large tumor size, and increased angiogenesis in breast cancer (Qin et al., 2006; Yamamoto et al., 2008; Li et al., 2018), which is consistent with the function of JFK. In light of the common view that tumor microenvironment is often hypoxic and HIF-1α is a hypoxia-inducible factor (Barker et al., 2015), we focused on the effect of HIF-1α on transcriptional regulation of *JFK.*

We further analyzed published chromatin immunoprecipitation-coupled massive parallel DNA sequencing (ChIP-seq) data for the genome-wide transcriptional profile of HIF-1α and to identify candidate direct transcriptional activation targets of HIF-1α in breast cancer (GSE59935) (Zhang et al., 2015). In these experiments, ChIP was performed in hypoxia-treated T47D cells using antibodies against HIF-1α (Zhang et al., 2015). After processing with the alignment to the unmasked human reference genome (GRCh37, hg19) using Bowtie2, we performed MACS2 (Model-based Analysis for ChIP-Seq) analysis using a cutoff of *q* < 1e-2. After filtering, a total of 99,681 HIF-1α-specific binding peaks were called, with the majority of the peaks located at promoter sequences (23.92%) and the remainder at intronic (32.71%) or intergenic (34.75%) regions (Figure 2C).

The genomic landscape of HIF-1α binding peaks at promoter sequences (≤3 K) were analyzed by deepTools (Figure 2D). Using R and Bioconductor, further GO enrichment analysis of the promoter regions (≤500 bp) with enriched HIF-1α occupancy revealed enrichment for functional annotations including mitotic cytokinesis, glycolytic process, cell cycle, and fatty acid β-oxidation, which were related to cell proliferation and metabolism (Figure 2E). Consistent with above prediction, tracks visualizing the ChIP signal analysis indicated that HIF-1α binds at the *JFK* promoter (Figure 2F). Quantitative ChIP (qChIP) analysis with antibodies against HIF-1α and *JFK*-specific primers of MCF-7 cells or T47D cells cultured in hypoxia (1% O_2_) conditions validated that the HIF-1α protein is highly enriched at *JFK* promoter (Figure 2G). Together, these observations suggest that *JFK* is a potential target gene of HIF-1α.

### *JFK* Is Transcriptionally Activated by HIF-1α

**FIGURE 3:**
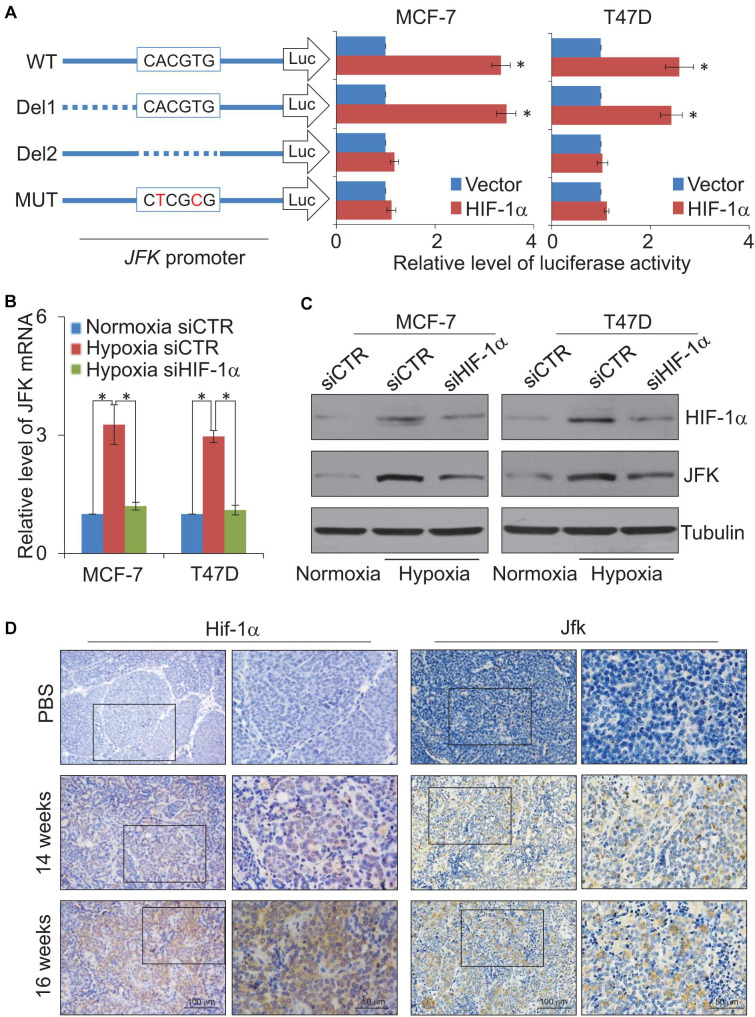
JFK is transcriptionally activated by HIF-1α. **(A)** MCF-7 or T47D cells infected with lentiviruses carrying *JFK*-Luc wild-type, deletion, or mutant variants, as well as control vector or HIF-1α expression constructs, were harvested, lysed, and assayed for firefly and Renilla luciferase activity using the dual-luciferase reporter assay system. Error bars represent the mean ± SD for triplicate experiments. **(B)** qPCR measurement of the *JFK* expression in MCF-7 or T47D cells treated with control or HIF-1α siRNAs, under normoxia or hypoxia (1% O_2_) for 24 h. Error bars represent the mean ± SD for triplicate experiments. **(C)** Western blotting analysis of cellular extracts from MCF-7 or T47D cells treated with control or HIF-1α siRNAs, under normoxia or hypoxia (1% O_2_) for 24 h. **(D)** Immunohistochemical staining of breast tumors from 14-week-old or 16-week-old *JFK*^*WT*^/*PyMT*^*TG*^ mice to assess the expression of Hif-1α and Jfk. Statistical significance of differences is indicated as *p < 0.05.

To further investigate the expression regulation of *JFK*, we next cloned a 600 bp fragment from the *JFK* promoter (Chr 1: 16,679,286–16,679,886) and constructed a *JFK* promoter-driven luciferase reporter (*JFK*-Luc). In these experiments, MCF-7 or T47D cells were co-transfected with HIF-1α expression plasmids together with the wild-type *JFK*-Luc promoter or a mutant *JFK*-Luc promoter lacking the predicted HIF-1α binding motif. The results showed that HIF-1α was able to activate transcription from the *JFK* promoter, but only when the CACGTG motif was present (Figure 3A), demonstrating that the HIF-1α protein can directly bind to the *JFK* promoter.

Both qPCR and western blotting were then performed in MCF-7 or T47D cells treated with control or HIF-1α siRNAs, under normoxia or hypoxia treatment for 24 h. The results showed that hypoxic conditions led to significantly increased expression of *JFK* (Figures 3B,C). No such increase in JFK mRNA or protein accumulation was observed upon HIF-1α knockdown in hypoxic condition (Figures 3B,C). These results demonstrate a direct functional impact of HIF-1α in the observed hypoxia-induced transcriptional activation of *JFK* in breast cancer cells.

Extending our investigation to the MMTV-PyMT mouse model, we harvested tumors from 14-week-old or 16-week-old *JFK*^*WT*^/*PyMT*^*TG*^ mice. Immunohistochemical staining showed that, compared to 14-week-old *JFK*^*WT*^/*PyMT*^*TG*^ mice, the Hif-1α and Jfk protein levels were clearly up-regulated in the mammary tumors of the 16-week-old *JFK*^*WT*^/*PyMT*^*TG*^ mice (Figure 3D). Collectively, these results support that the hypoxia-mediated induction of JFK is mediated by HIF-1α binding and transcriptional activation and, further, defines an HIF-1α-JFK axis which apparently functions in promoting breast carcinogenesis.

### JFK Promotes HIF-1α-Induced Glycolysis

**FIGURE 4:**
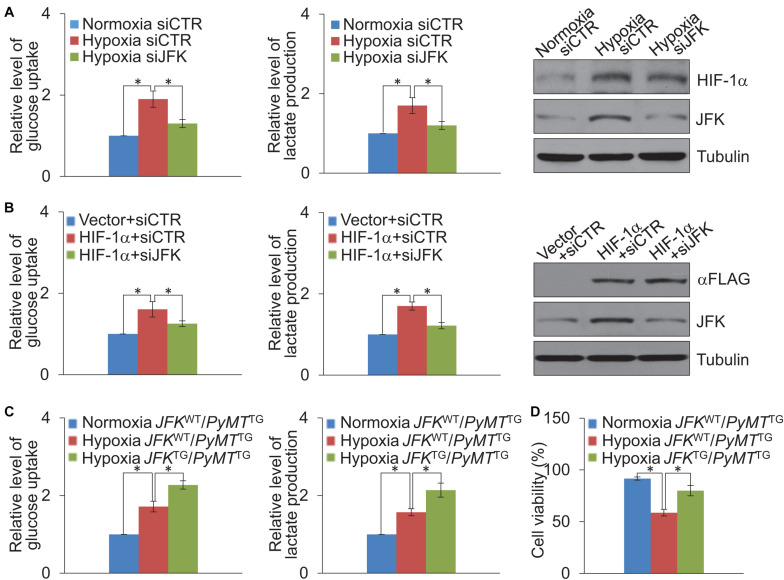
JFK promotes HIF-1α-induced glycolysis. **(A)** Cellular extracts or cell culture medium of MCF-7 cells treated with control or JFK siRNAs and cultured in hypoxia (1% O_2_) conditions for 24 h were prepared for measurement of intracellular glucose or extracellular lactate. Error bars represent the mean ± SD for triplicate experiments. Cellular extracts were prepared for western blotting analysis with the indicated antibodies. **(B)** Cellular extracts or cell culture medium of MCF-7 cells treated with control or JFK siRNAs and transfected with vector or HIF-1α were prepared for measurement of intracellular glucose or extracellular lactate. Error bars represent the mean ± SD for triplicate experiments. Cellular extracts were prepared for western blotting analysis with the indicated antibodies. **(C)** Cellular extracts or cell culture medium of primary tumor cells from *JFK*^*WT*^/*PyMT*^*TG*^ or *JFK*^*TG*^/*PyMT*^*TG*^ mice cultured in normoxia or hypoxia (1% O_2_) conditions for 24 h were prepared for measurement of intracellular glucose or extracellular lactate. Error bars represent the mean ± SD for triplicate experiments. **(D)** Primary tumor cells from *JFK*^*WT*^/*PyMT*^*TG*^ or *JFK*^*TG*^/*PyMT*^*TG*^ mice cultured in normoxia or hypoxia (0.2% O_2_) conditions for 72 h, followed by cell viability assessment. Error bars represent the mean ± SD for three independent experiments. Statistical significance of differences is indicated as *p < 0.05.

It is known that HIF-1α is a metabolic regulator that plays an important role in glycolysis metabolism (Wu et al., 2020), we further investigated whether JFK functions in hypoxia-induced glycolysis of breast cancer cells. To this end, MCF-7 cells treated with control or JFK siRNAs were cultured in normoxia or hypoxia conditions, or co-transfected with vector or HIF-1α, and we performed glucose colorimetric/fluorometric or lactate colorimetric assay to detect glucose uptake or lactate production. The results showed that JFK knockdown significantly dampened hypoxia or HIF-1α-induced glycolysis in breast cancer cells (Figures 4A,B).

To further extend our investigation to the MMTV-PyMT mouse model, we digested tumor tissues from *JFK*^*WT*^/*PyMT*^*TG*^ or *JFK*^*TG*^/*PyMT*^*TG*^ mice, and the isolated cells were put into primary culture. Isolated cells cultured in normoxia or hypoxia conditions were performed glucose colorimetric/fluorometric or lactate colorimetric assay to detect glucose uptake or lactate production. The results revealed that JFK overexpression significantly strengthened hypoxia-induced glycolysis in breast cancer (Figure 4C). Furthermore, primary tumor cells were cultured in normoxia or hypoxia conditions for 72 h, and cell viability analysis showed that the JFK overexpression significantly weakened the decreased cell viability under hypoxia (Figure 4D). Together, these results suggest that *JFK* promotes HIF-1α-induced glycolysis thus elevating cellular tolerance to hypoxia in breast cancer.

### JFK Deficiency Sensitizes Hypoxic Breast Cancer Cells to Chemo-Radiotherapeutic Treatment

**FIGURE 5:**
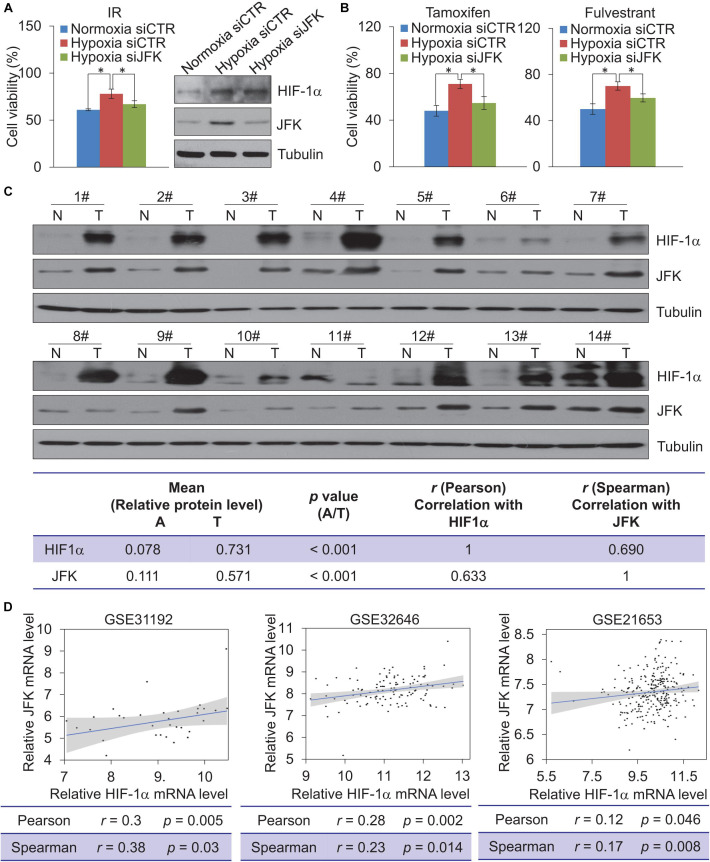
JFK deficiency sensitizes hypoxic breast cancer cells to chemo-radiotherapeutic treatment. **(A)** MCF-7 cells treated with control or JFK siRNAs were cultured in normoxia or hypoxia (1% O_2_) conditions, and treated with IR (5 Gy) and subjected to cell viability assessment. Error bars represent the mean ± SD for triplicate experiments. Cellular extracts were prepared for western blotting analysis with the indicated antibodies. **(B)** MCF-7 cells treated with control or JFK siRNAs were cultured in normoxia or hypoxia (1% O_2_) conditions, and treated with tamoxifen (0.5 μM) or fulvestrant (0.5 μM) and subjected to cell viability assessment. Error bars represent the mean ± SD for triplicate experiments. **(C)** Western blotting analysis in 14 breast tumors (T) paired with adjacent normal tissues (N) from breast carcinoma patients for the expression of HIF-1α and JFK. Quantitation was performed based on densitometry using Image J, and the values are expressed after normalization to the level of tubulin. The results from correlation analysis between HIF-1α and JFK expression are shown. **(D)** The level of *JFK* mRNA expression was plotted against the level of *HIF-1α* mRNA expression in published clinical datasets (GSE31992, GSE32646, and GSE21653); the line presents a fitted linear model, and the shading represents the 95% confidence interval. Statistical significance of differences is indicated as *p < 0.05.

Considering that the high expression of HIF-1α has been related to resistance to radiotherapy and chemotherapy in breast cancer (Yang J. et al., 2015), we next investigated the role of JFK in breast cancer cell sensitivity to ionizing radiation (IR) and chemotherapeutic agents under hypoxia. To this end, MCF-7 cells treated with control or JFK siRNAs under normoxia or hypoxia were exposed to X-ray irradiation or treated with chemotherapeutic agents (tamoxifen or fulvestrant). Cell viability analysis showed that JFK deficiency significantly weakened the increased cell viability in response to hypoxia-induced resistance to IR or chemotherapeutic agents of MCF-7 cells (Figures 5A,B). These observations suggest that JFK protected cells from toxic insults and promoted cell survival under hypoxia.

To gain further support for the impacts of this HIF-1α-JFK axis on breast cancer, and seeking to add clinicopathologically relevant data to our observations, we collected samples from resected breast carcinomas – paired with adjacent normal mammary tissues – from 14 breast cancer patients. Western blotting analysis showed that both HIF-1α and JFK protein levels were significantly upregulated in the cancer tissue compared to the normal tissue (Figure 5C). Additionally, and consistent with HIF-1α as a transcription activator of *JFK*, quantitation and statistical analysis revealed that the relative level of JFK protein was significantly and positively correlated with that of HIF-1α (Spearman’s rank correlation coefficient, 0.690; *p* < 0.001) (Figure 5C).

We also analyzed public data to evaluate the *HIF-1*α and *JFK* mRNA expression levels in breast cancers. Analysis of published clinical datasets (GSE31192, GSE32646, and GSE21653) revealed a positive correlation between *HIF-1*α and *JFK* mRNA expression detected for all of the examined datasets (Figure 5D). At minimum, these analyses of previously published breast cancer datasets and our own analysis of tissues from 14 breast cancer patients support our identification of the HIF-1α-JFK axis. Collectively, these findings support a notion that HIF-1α-JFK axis is an important regulator that acts to elevate cellular tolerance to hypoxia and contribute to cell survival in promoting breast carcinogenesis.

## Discussion

Elevated accumulation of the HIF-1α protein has been observed in a broad array of human cancer cell types including breast cancer cells, in which elevated levels of HIF-1α are associated with poor prognosis (Jun et al., 2017). Specifically, knockout of *HIF-1*α in the MMTV-PyMT mouse model was reported to suppress both primary tumor growth and lung metastasis (Schwab et al., 2012), which is consistent with our observation that *JFK* overexpression promotes mammary tumor initiation and metastasis in the MMTV-PyMT mouse model. Together these findings support our hypothesis that the HIF-1α-JFK axis contributes to breast cancer cell survival. Our observation of selective (i.e., hypoxia-specific) transcriptional activation of *JFK* by HIF-1α is notable because JFK dysregulation/overexpression has been reported to be a frequent event in breast cancer and has been associated with especially aggressive clinical features and worse survival in breast cancer patients (Yan et al., 2015).

The HREs bound by HIF-1α and HIF-2α have both been shown to contain a conserved RCGTG core sequence, and there are reports that they share transcription target genes including *IL-6*, *FILAG*, and *GLUT1*, which are involved in the adaptation of tumor cells to hypoxia (Keith et al., 2011); thus, it is possible that HIF-2α may also bind to the HRE in the *JFK* promoter. A previous study reported that the HIF-2α protein is stabilized under 5% O_2_ (resembling end capillary oxygen conditions) and – in contrast to the low transcription activation activity of HIF-1α at this oxygen level – HIF-2α strongly promotes the transcription of genes such as *VEGF* (Holmquist-Mengelbier et al., 2006). It is known that untreated breast cancer tumors can be hypoxic (∼1.3% oxygen); this level is >5 times lower than oxygen levels found in the normal breast tissues (Wang et al., 2015; Rankin and Giaccia, 2016). Given that O_2_ levels vary widely across sub-domains of solid tumors [owing, for example, to rapid cell division and aberrant tumor angiogenesis and blood flow (Bertout et al., 2008)], it will be interesting to explore whether *JFK* is a co-target of both HIF-1α and HIF-2α, perhaps under different severities of hypoxia, in different tumor sub-domains, or at different stages of breast cancer development. Clarifying these points should deepen our understanding of the roles of hypoxia generally, and JFK and HIF-1α/HIF-2α specifically, in breast cancer.

Among breast cancer tumors, basal-like tumors are the most aggressive and are associated with the highest rates of metastasis and recurrence (Alluri and Newman, 2014). Clinical dataset analyses in our previous study revealed that higher *JFK* expression levels were associated with worse overall survival in basal-like breast cancer patients (Yan et al., 2015), so it will be interesting to further investigate whether *JFK* levels in, for example, diagnostic tumor biopsies may be useful for identifying basal-like breast cancers in patients, which are at increased risk of developing metastasis. Consistently, HIF-1α has been identified as one of five signature markers predictive of disease outcomes among node-negative breast cancer patients (Charpin et al., 2012). In addition, it is known that basal-like tumors frequently show morphologic evidence of hypoxia (central fibrosis and necrosis) (Livasy et al., 2006); conventional chemotherapy is not always effective on these tumors and a systemic relapse is often observed, potentially due to the development of drug resistance (Boichuk et al., 2017). Our study revealed that JFK deficiency sensitizes breast cancer cells to chemo-radiotherapeutic treatment under hypoxia, indicating that hypoxia-induced JFK upregulation in basal-like tumors might contribute to drug resistance.

Obesity has been proposed to be a highly significant risk factor for breast carcinogenesis and aggressiveness (Perez-Hernandez et al., 2014), as excessive adipose tissue in obese patients not only alters the local microenvironment by remodeling the extracellular matrix but also provides both energy and niches for cancer cell proliferation and invasion (Seo et al., 2015; Gao et al., 2020). In this regard, drugs (e.g., metformin) targeting signaling pathways that are deregulated in obesity might help to prevent carcinogenesis in obese women (Oppong et al., 2014; Gao et al., 2020). Intriguingly, our recent study demonstrated that JFK suppresses hepatic lipid catabolism and exacerbates the development of obesity and non-alcoholic fatty liver disease (NAFLD) by destabilizing ING5. Moreover, *JFK* is up-regulated in liver tissues from NAFLD patients (*n* = 206) as compared with healthy controls (*n* = 10) according to a recently published public dataset (GSE135251) (Govaere et al., 2020). In the present study, we show that JFK promotes mammary progression in mice, and that the protein level of JFK is up-regulated in resected breast tumors compared with the adjacent normal tissues from breast cancer patients. Although the molecular mechanisms underlying the strong association between obesity and poor breast cancer outcomes are not well understood, it seems plausible that JFK acts as a regulatory hub that coordinates obesity and breast carcinogenesis. Considering that HIF-1α controls oxygen delivery into tumors via angiogenesis and metabolic adaptation of tumors to hypoxia via glycolysis (Semenza, 2002), the HIF-1α-JFK axis we identified in the present study may regulate processes (including glycolysis and fatty-acid β-oxidation) with broad impacts on tumor initiation and metastasis. Moreover, further exploratory screens for interaction partners or degradation substrates of JFK under several conditions including hypoxia, dysregulated energy metabolism, and lipid abnormalities to characterize how JFK functions in apparently diverse cellular processes would likely be scientifically informative.

Our previous studies also revealed that JFK depletion suppresses proliferation and angiogenesis of breast cancer cells through accumulation of tumor suppressors p53 and ING4 (Sun et al., 2009, 2011; Yan et al., 2015). In this study, we showed that JFK depletion reduces cellular tolerance to hypoxia by suppressing glycolysis, and thus contributes to cell survival in breast cancer, which deepens the mechanistic understanding of cancer cell responses to normoxic versus hypoxic conditions. The fact that cancer cells can become addicted to specific metabolic pathways has led to the recent development of novel drugs that target these metabolic vulnerabilities (Tennant et al., 2010). Beyond defining the hypoxia-sensitive HIF-1α-SCF^*JFK*^ axis, which promotes breast carcinogenesis, we extended these molecular and cell-context specific insights into clinical relevance when targeting HIF-1α is not effective, supporting the hypothesis that JFK is a vulnerable target for the development of innovative therapeutic interventions against breast cancer.

## Data Availability Statement

The datasets presented in this study can be found in online repositories. The names of the repository/repositories and accession number(s) can be found in the article/Supplementary Material.

## Ethics Statement

The animal study was reviewed and approved by the Institutional Animal Care and Use Committee at Peking University.

## Author Contributions

ZY, LH, and LS designed the research. ZY, XZ, and LH conducted the experiments. ZY, XZ, LH, YpW, EZ, and YZ performed the animal experiments and analyzed the data. YzW, XL, YW, ZL, FP, JR, YH, LX, SG, SQ, FS, JS, and LW provided technical assistance. ZY, XZ, LH, and LS wrote the manuscript. All authors contributed to the article and approved the submitted version.

## Conflict of Interest

The authors declare that the research was conducted in the absence of any commercial or financial relationships that could be construed as a potential conflict of interest.

## Footnotes

1 http://alggen.lsi.upc.es/cgi-bin/promo_v3/promo/promoinit.cgi?dirDB=TF_8.3

2 http://phenolyzer.wglab.org/

## Supplementary Material

Supplementary Movie 1 Three-dimensional SPECT/CT image of *JFK*^*WT*^/*PyMT*^*TG*^ at 30 min after intravenous injection of ^99^mTc-3PRGD2.

Supplementary Movie 2 Three-dimensional SPECT/CT image of *JFK*^*TG*^/*PyMT*^*TG*^ at 30 min after intravenous injection of ^99^mTc-3PRGD2.
